# Nitroxyl (HNO) Stimulates Soluble Guanylyl Cyclase to Suppress Cardiomyocyte Hypertrophy and Superoxide Generation

**DOI:** 10.1371/journal.pone.0034892

**Published:** 2012-04-10

**Authors:** Eliane Q. Lin, Jennifer C. Irvine, Anh H. Cao, Amy E. Alexander, Jane E. Love, Ruchi Patel, Julie R. McMullen, David M. Kaye, Barbara K. Kemp-Harper, Rebecca H. Ritchie

**Affiliations:** 1 Baker IDI Heart and Diabetes Institute, Melbourne, Australia; 2 Department of Pharmacology, Monash University, Clayton, Victoria, Australia; 3 Department of Physiology, Monash University, Clayton, Victoria, Australia; 4 Department of Medicine, Monash University, Clayton, Victoria, Australia; Ohio State University, United States of America

## Abstract

**Background:**

New therapeutic targets for cardiac hypertrophy, an independent risk factor for heart failure and death, are essential. HNO is a novel redox sibling of NO• attracting considerable attention for the treatment of cardiovascular disorders, eliciting cGMP-dependent vasodilatation yet cGMP-independent positive inotropy. The impact of HNO on cardiac hypertrophy (which is negatively regulated by cGMP) however has not been investigated.

**Methods:**

Neonatal rat cardiomyocytes were incubated with angiotensin II (Ang II) in the presence and absence of the HNO donor Angeli's salt (sodium trioxodinitrate) or B-type natriuretic peptide, BNP (all 1 µmol/L). Hypertrophic responses and its triggers, as well as cGMP signaling, were determined.

**Results:**

We now demonstrate that Angeli's salt inhibits Ang II-induced hypertrophic responses in cardiomyocytes, including increases in cardiomyocyte size, *de novo* protein synthesis and β-myosin heavy chain expression. Angeli's salt also suppresses Ang II induction of key triggers of the cardiomyocyte hypertrophic response, including NADPH oxidase (on both Nox2 expression and superoxide generation), as well as p38 mitogen-activated protein kinase (p38MAPK). The antihypertrophic, superoxide-suppressing and cGMP-elevating effects of Angeli's salt were mimicked by BNP. We also demonstrate that the effects of Angeli's salt are specifically mediated by HNO (with no role for NO• or nitrite), with subsequent activation of cardiomyocyte soluble guanylyl cyclase (sGC) and cGMP signaling (on both cGMP-dependent protein kinase, cGK-I and phosphorylation of vasodilator-stimulated phosphoprotein, VASP).

**Conclusions:**

Our results demonstrate that HNO prevents cardiomyocyte hypertrophy, and that cGMP-dependent NADPH oxidase suppression contributes to these antihypertrophic actions. HNO donors may thus represent innovative pharmacotherapy for cardiac hypertrophy.

## Introduction

Cardiac hypertrophy is strongly implicated in the development of heart failure of almost all etiologies. In addition to heart failure, it remains an independent risk factor for myocardial infarction and sudden death [1]–[3]. Cardiac hypertrophy initially develops *in vivo* as an adaptive response to maintain myocardial function, for example in hypertension when cardiac workload is chronically elevated [4]. Individual cardiomyocytes hypertrophy, accompanied by re-expression of embryonic genes, a switch in prevalence of contractile protein expression from α- to β-myosin heavy chain and sarcomeric organization [2], [3]. Ultimately, hypertrophy may progress to a maladaptive state, with progressive decline in ventricular contractility and diastolic function, with adverse outcomes [4], [5]. Current therapies (e.g. renin-angiotensin system inhibition) slow progression of cardiac hypertrophy, but patients still die with enlarged hearts. Identification of new therapeutic targets to prevent or reverse cardiac hypertrophy is essential [3].

We and other have shown that the nitroxyl anion, NO^−^, the one electron reduction product of NO^•^, is a novel regulator of cardiovascular function [3], [6]–[16]. At physiological pH, nitroxyl exists predominantly in the protonated form as HNO [12]. Similar to NO^•^, HNO mediates potent vasodilatation, largely via sGC activation and an elevation in cGMP [6], [7]. In direct contrast to NO^•^ however, HNO also elicits a marked inotropic effect (independent of cGMP), that persists even in failing myocardium *in vivo* [3], [8], [9]. Other distinct advantages offered by HNO include its lack of reactivity with reactive oxygen species (ROS) [14], [17]–[19], an absence of tolerance development [15], [20] and a direct interaction with thiols [10]–[12]. Much of this evidence has been obtained using the HNO donor, Angeli's salt (sodium trioxodinitrate, Na_2_N_2_O_3_), which releases both HNO and nitrite [3], [12]. Cardiac HNO actions (in contrast to those of NO^•^) may thus well be preserved under conditions of oxidative stress (e.g. cardiac hypertrophy and heart failure) [3], [13]. With these therapeutic advantages, HNO donors are now in development for clinical management of acute heart failure events.

cGMP-dependent signaling is a powerful antihypertrophic and ROS-suppressing mechanism in the heart; much of this work has emanated from our own studies [3], [21]–[28]. Exploiting cGMP for the treatment of hypertrophy and heart failure via conventional NO^•^ donors is limited however by the rapid reaction of ROS with NO^•^ to form peroxynitrite and impairing NO^•^ bioavailability [3]. Given the ability of HNO to stimulate sGC even in settings of elevated ROS, we now test the hypothesis that HNO elicits cGMP-dependent antihypertrophic effects in neonatal rat cardiomyocytes. Further, given that HNO elicits antioxidant actions in yeast, cell-free systems and in vascular tissues [13], [29], the impact on cardiomyocyte NADPH oxidase was also determined. BNP, a cGMP-elevating agent with known antihypertrophic efficacy [21], [24], [26], was used for comparison. Our results provide the first evidence that the HNO donor Angeli's salt prevents cardiomyocyte hypertrophy, and that cGMP-dependent suppression of cardiomyocyte NADPH oxidase contributes to these antihypertrophic actions.

## Materials and Methods

This investigation conforms with both the *Guide for the Care and Use of Laboratory Animals* published by the US National Institutes of Health (NIH Publications No. 85-23, revised 1996) and the National Health and Medical Research Council of Australia guidelines, and was approved by the Alfred Medical, Research and Education Precinct (AMREP) Animal Ethics Committee (approval E/0698/2008/B). All materials were purchased from Sigma-Aldrich (St. Louis, USA) except where indicated, and were of analytic grade or higher.

### Hypertrophic responses in primary neonatal rat cardiomyocytes

Hearts were collected from 1- to 2-day-old neonatal rat pups, promptly after euthanasia by decapitation. Cardiomyocytes were then isolated, and plated at a density of 1×10^3^ cells/mm^2^ for determination of all measures except two-dimensional (2D) cardiomyocyte size, in which cells were plated at a density of 2×10^2^ cells/mm^2^ (to permit delineation of defined single cells, as previously described) [21]. All materials used for cardiomyocyte isolation were of tissue culture grade. Following 48 h incubation under serum-free conditions, cardiomyocytes were incubated for 48 h in the presence and absence of the hypertrophic stimuli angiotensin II (Ang II, 1 µmol/L, Auspep, Parkville, Australia) or endothelin-1 (ET_1_, 60 nmol/L) [21], [26], [30], and/or the HNO donor Angeli's salt (sodium trioxodinitrate, 1 µmol/L unless otherwise stated [6], added 4×/day to compensate for its shorter half-life, Cayman chemicals, Michigan, USA). BNP (1 µmol/L, Auspep) and the stable cGMP analog 8-bromo-cGMP (8BrcGMP, 1 mmol/L) were used for comparison [21], [26]. Concentrations of all drugs studied were based on those previously reported, as indicated. The vehicle control for Angeli's salt, 0.01 mol/L NaOH [15], was incorporated into the study design, and was also added 4×/day. Markers of cardiomyocyte hypertrophy included 2D area (µm^2^) of live cells (30 individual myocytes measured per treatment), *de novo* protein synthesis (determined via incorporation of [^3^H]phenylalanine, Amersham Biosciences, Castle Hill, Australia), 4 replicates per treatment), and expression of the pro-hypertrophic gene, β-myosin heavy chain, as previously described [21], [22], [26], [30]. Real time PCR reagents were all of molecular biology grade, and included Taqman® reverse transcription reagents, Taqman® Universal PCR master mix, DNase treatment kits, fluorogenic probes (Applied Biosystems, Scoresby, Australia), as well as forward and reverse primers for real-time PCR (Geneworks, Thebarton, Australia).

### Triggers of cardiomyocyte hypertrophy

The impact of Angeli's salt on key triggers of pathological hypertrophy included cardiomyocyte expression of the Nox2 subunit of NADPH oxidase, superoxide generation, and phosphorylation of p38MAPK, as previously described [21], [31]. In addition, phosphorylation of the cell survival kinase Akt and its downstream target glycogen synthase kinase-3β (GSK-3β, as well as of the mitogen-activated protein kinase ERK1/2, were also determined [30]. For determination of Nox2 expression, cells were incubated for 48 h in the presence and absence of Ang II or ET_1_, and/or Angeli's salt (replenished 4×/day). Relative quantification of changes in cardiomyocyte expression of the Nox2 subunit of NADPH oxidase (a major source of ROS), was determined using real time PCR analysis, with 18S as the endogenous control, as previously described [21], [31]. Cardiomyocyte superoxide generation was determined using NADPH-driven lucigenin-enhanced chemiluminescence, an estimate of NADPH oxidase activity, as previously described [21], [31], [32]. Cells were incubated for 48 h in the presence or absence of Angeli's salt, BNP, 8BrcGMP, with Ang II or ET_1_, added for the final 24 h. Each measurement was expressed as relative light units per second (RLU/sec). Background luminescence (in the absence of cells) was subtracted from the average of 8 readings. Each experiment was studied with at least 4 replicates, and the average result was taken. In a separate series of experiments, cardiomyocyte activation of the mitogen-activated protein kinases ERK1/2 and p38MAPK, as well as phosphorylation of Akt and glycogen synthase kinase-3β (GSK-3β, were determined in the presence or absence of Angeli's salt for 48 h; Ang II was added only for the final 10 min. Western analyses used phospho-specific antibodies (Cell Signaling Technology, Danvers, MA), as previously described [30], [32].

### HNO/sGC/cGMP signaling

The role of sGC and cGK-I in mediating the actions of Angeli's salt in cardiomyocytes was determined using the selective inhibitors, ODQ (1 µmol/L) [15] and KT5823 (250 nmol/L, Calbiochem-Novabiochem, La Jolla, CA) [21], [26], respectively. The vehicle control for KT5823 and ODQ (0.01% DMSO) was also incorporated into study design. The impact of Angeli's salt on cardiomyocyte protein levels of cGK-I and sGC (48 h incubation), and phosphorylation of VASP (10 min incubation, a biomarker of cGK-I signaling) were determined, via Western analysis, using primary antibodies from Cell Signaling Technology. Cell-free purified sGC activity was determined by conversion of GTP (40 µmol/L, Sigma) to cGMP by sGC (34 ng, Alexis Biochemicals, San Diego, CA) over 10 min, in the presence and absence of Angeli's salt [33]. Cardiomyocyte cGMP generation was also, determined via enzyme immunoassay (Cayman Chemical) following 5 and 15 min incubation with either Angeli's salt or BNP, as previously described [21].

The relative roles of HNO and NO^•^ in the actions of Angeli's salt were determined firstly on generation of NO^•^ using an NO^•^-sensing electrode (World Precision Instruments, Sarasota, FL) in the presence and absence of Angeli's salt, and results compared to the pure NO^•^ donor, DEA/NO (both 0.1–30 µmol/L, Cayman Chemical) [15]. Subsequent studies used the selective scavengers, L-cysteine (3 mmol/L, for HNO) and 2-(4-carboxyphenyl)-4,4,5,5-tetramethylimidazoline-1-oxyl-3-oxide(carboxy-PTIO, 200 µmol/L, for NO^•^) [15]. In addition, the potential cGMP-elevating and antihypertrophic effects of both sodium nitrite (1 µmol/L) and degraded Angeli's salt (1 µmol/L, replenished 4×/day, obtained by storing Angeli's salt solution at room temperature for 48 h, followed by 2 h at 37°C, prior to use), was determined.

### Statistical analysis

All results were expressed as mean ± standard error for each treatment group, with the number of myocyte preparations studied denoted by “n”. Changes in [^3^H]phenylalanine incorporation, 2D cardiomyocyte size, superoxide generation and cGMP content were expressed as a percentage of paired control cardiomyocytes from the same preparation. For changes in both gene expression (β-myosin heavy chain, Nox2) and protein (ERK1/2, p38MAPK, Akt, GSK-3β, sGC, cGK-I, P-VASP), results were expressed as a fold of paired control. Statistical comparison of ≥3 different experimental groups was performed using one way repeated measures analysis of variance to compare the effect of Ang II with paired control, or of antihypertrophic interventions (e.g. Angeli's salt in the presence of Ang II) with Ang II alone, where n≥4. The Student-Newman-Keuls correction for pairwise multiple comparisons was applied where required. Where the experiment only used 2 groups, the effect of Angeli's salt alone versus control was compared using paired *t*-tests. Results with P values<0.05 were considered statistically significant.

## Results

### Antihypertrophic actions of Angeli's salt in neonatal cardiomyocytes

**Figure 1:**
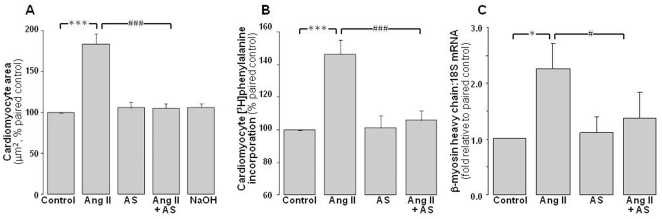
Antihypertrophic actions of Angeli's salt. Ang II (1 µmol/L, 48 h)-stimulated cardiomyocyte hypertrophy is abolished by Angeli's salt (AS, 1 µmol/L, added 4×/day over 48 h). This is evident on **A** cardiomyocyte area (n = 10 myocyte preparations); **B** *de novo* protein synthesis (on [^3^H]phenylalanine incorporation, n = 9 myocyte preparations); and **C** hypertrophic gene expression (using the fetal isoform of the contractile protein, β-myosin heavy chain, n = 6 myocyte preparations). *P<0.05 and ***P<0.001 vs control; ^#^P<0.05 and ^###^P<0.001 vs Ang II alone.

The hypertrophic stimulus, Ang II (1 µmol/L), induces hypertrophic responses in neonatal rat cardiomyocytes, increasing 2D area to 184±12% of control (Figure 1A, P<0.001), *de novo* protein synthesis ([^3^H]phenylalanine incorporation) to 146±9% (Figure 1B, P<0.001), and β-myosin heavy chain expression to 2.3±0.4-fold of paired control myocytes (Figure 1C, P<0.05). The HNO donor Angeli's salt (1 µmol/L, added 4×/day over 48 h) exerts marked anti-hypertrophic actions, virtually abolishing the Ang II-induced increases in 2D area (Figure 1A, P<0.001 vs Ang II alone), protein synthesis (Figure 1B, P<0.001 vs Ang II alone) and hypertrophic gene expression (Figure 1C, P<0.05 vs Ang II alone). Angeli's salt alone does not significantly affect 2D area, protein synthesis or β-myosin heavy chain in neonatal cardiomyocytes. Further, as shown in Table 1, the NaOH vehicle used for Angeli's salt does not significantly affect cardiomyocyte responses, either alone or in the presence of Ang II (Table 1).

**Table 1:** NaOH (0.01 mol/L), the vehicle used for Angeli's salt, does not affect neonatal rat cardiomyocyte responses, alone or in the presence of Ang II (1 µmol/L).

	Control	NaOH	Ang II	Ang II+NaOH	n
**Cell size**	100±0%	109±7%	131±9%**	125±2%*	7
**Protein synthesis**	100±0%	107±10%	142±9%*	143±7%*	3
**Superoxide**	100±0%	159±22%	233±43%*	212±33%*	4

* P<0.05 and

** P<0.01 vs control.

### Angeli's salt suppresses ROS generation in neonatal cardiomyocytes

**Figure 2:**
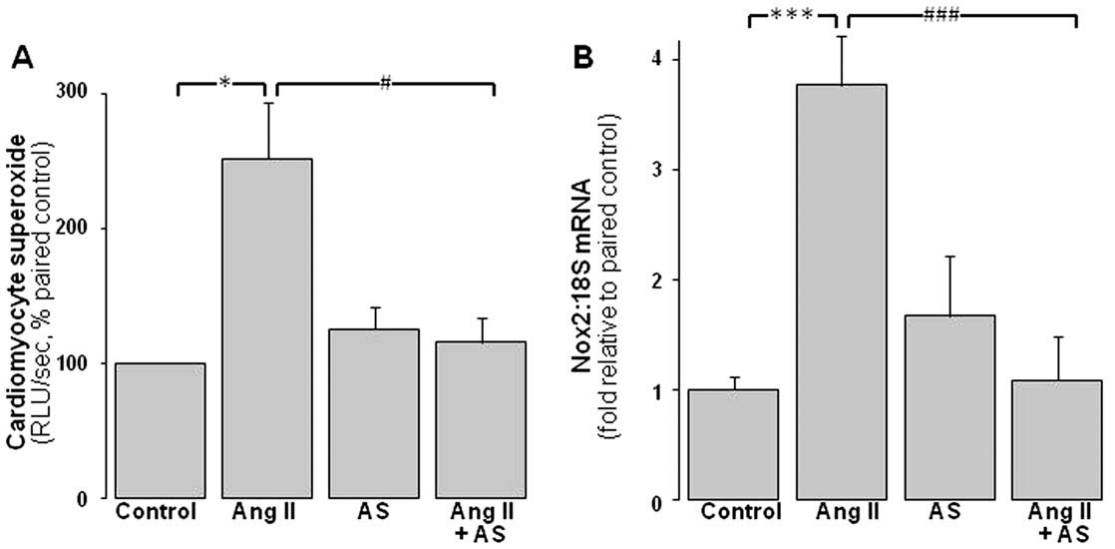
ROS-suppressing actions of Angeli's salt. AS (1 µmol/L, added 4×/day over 48 h) blocks cardiomyocyte NADPH oxidase activity and expression. **A** Ang II (1 µmol/L, final 24 h)-induced superoxide generation (lucigenin chemiluminescence, n = 11 myocyte preparations); **B** Ang II (1 µmol/L, 48 h)-induced cardiomyocyte Nox2 gene expression (n = 8 myocyte preparations). *P<0.05 and ***P<0.001 vs control; ^#^P<0.05 and ^###^P<0.001 vs Ang II alone.

Ang II significantly increases NADPH-driven cardiomyocyte superoxide generation 2.5±0.4 fold paired control (Figure 2A, P<0.05). Angeli's salt completely prevents Ang II induction of cardiomyocyte superoxide (Figure 2A, P<0.05 vs Ang II alone). Ang II-induced increases in Nox2 expression (to 3.8±0.5-fold of control) are also completely abolished by Angeli's salt (Figure 2B, P<0.001 vs Ang II alone). Angeli's salt alone does not significantly affect either parameter.

### Impact of Angeli's salt on cardiomyocyte pro-growth signaling

**Figure 3:**
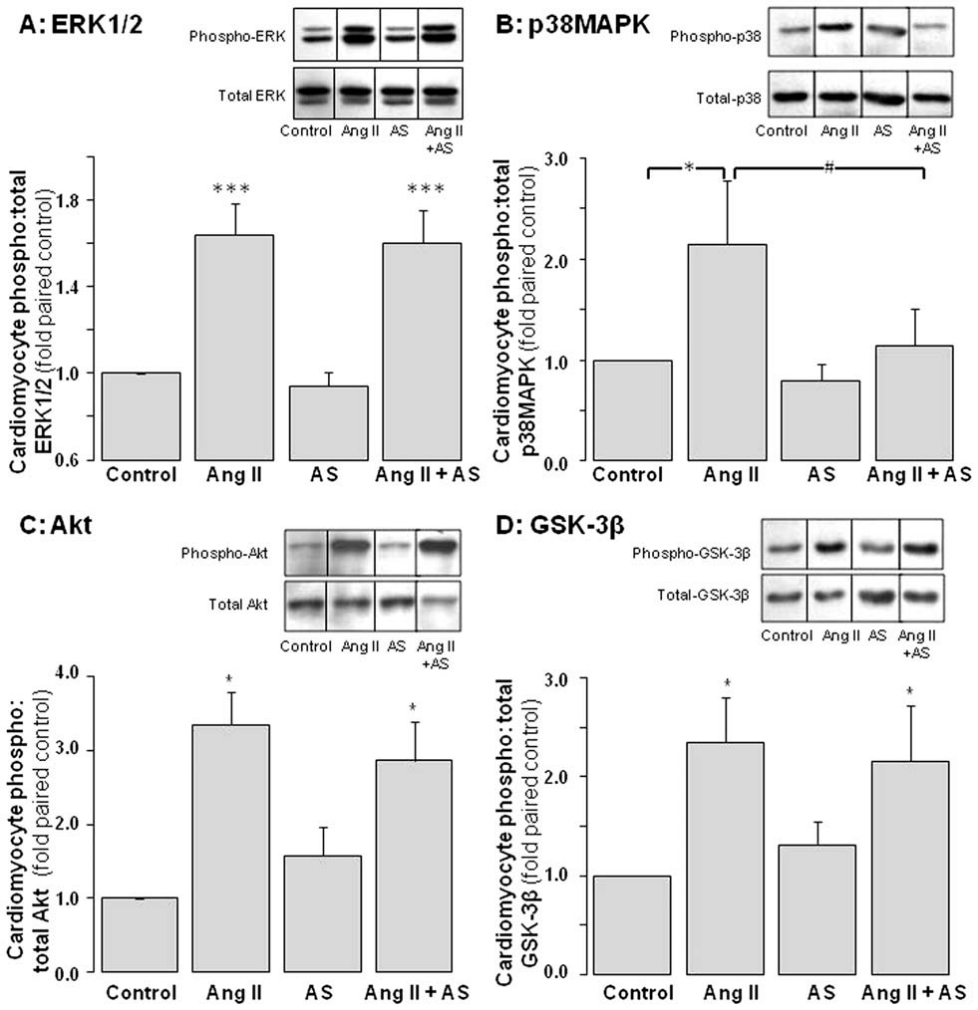
Impact of Angeli's salt on cardiomyocyte pro-hypertrophic signaling. AS (1 µmol/L, added 4×/day over 48 h) selectively inhibits Ang II (1 µmol/L, final 10 min)-stimulated p38MAPK phosphorylation of pro-hypertrophic signaling. Ang II-stimulated phosphorylation of ERK1/2 and Akt (and its downstream target GSK-3β) are preserved. Phosphorylation of **A** ERK1/2 (n = 8 myocyte preparations); **B** p38MAPK (n = 7 myocyte preparations); **C** Akt (n = 5 myocyte preparations, P<0.05); and **D** GSK-3β (n = 9 myocyte preparations, P<0.01), all as a ratio of total kinase. Representative images for phospho- and total kinases (from the same blot) are shown in the inset of each panel. *P<0.05 and ***P<0.001 vs control; ^#^P<0.05 and ^###^P<0.001 vs Ang II alone.

Ang II (1 µmol/L) activates several pro-hypertrophic signals in cardiomyocytes, including ERK1/2 (by 1.6±0.1-fold, Figure 3A, P<0.001), p38MAPK (2.1±0.6-fold, Figure 3B, P<0.05), Akt (3.4±0.4-fold, Figure 3C, P<0.05), and p70S6-kinase (by 2.0±0.2-fold, n = 4 cardiomyocyte preparations, P<0.05). Ang II also decreases activity of GSK-3β (an Akt-sensitive negative regulator of hypertrophy), as indicated by increased GSK-3β phosphorylation (by 2.3±0.4-fold, Figure 3D, P<0.05). Pretreatment with Angeli's salt (1 µmol/L) significantly attenuated Ang II-mediated p38MAPK activation (P<0.05 versus Ang II alone), without significant impact on Ang II-mediated ERK1/2 or Akt activation, or GSK-3β phosphorylation (Figure 3).

### The antihypertrophic actions of Angeli's salt utilize sGC/cGMP signaling

**Figure 4:**
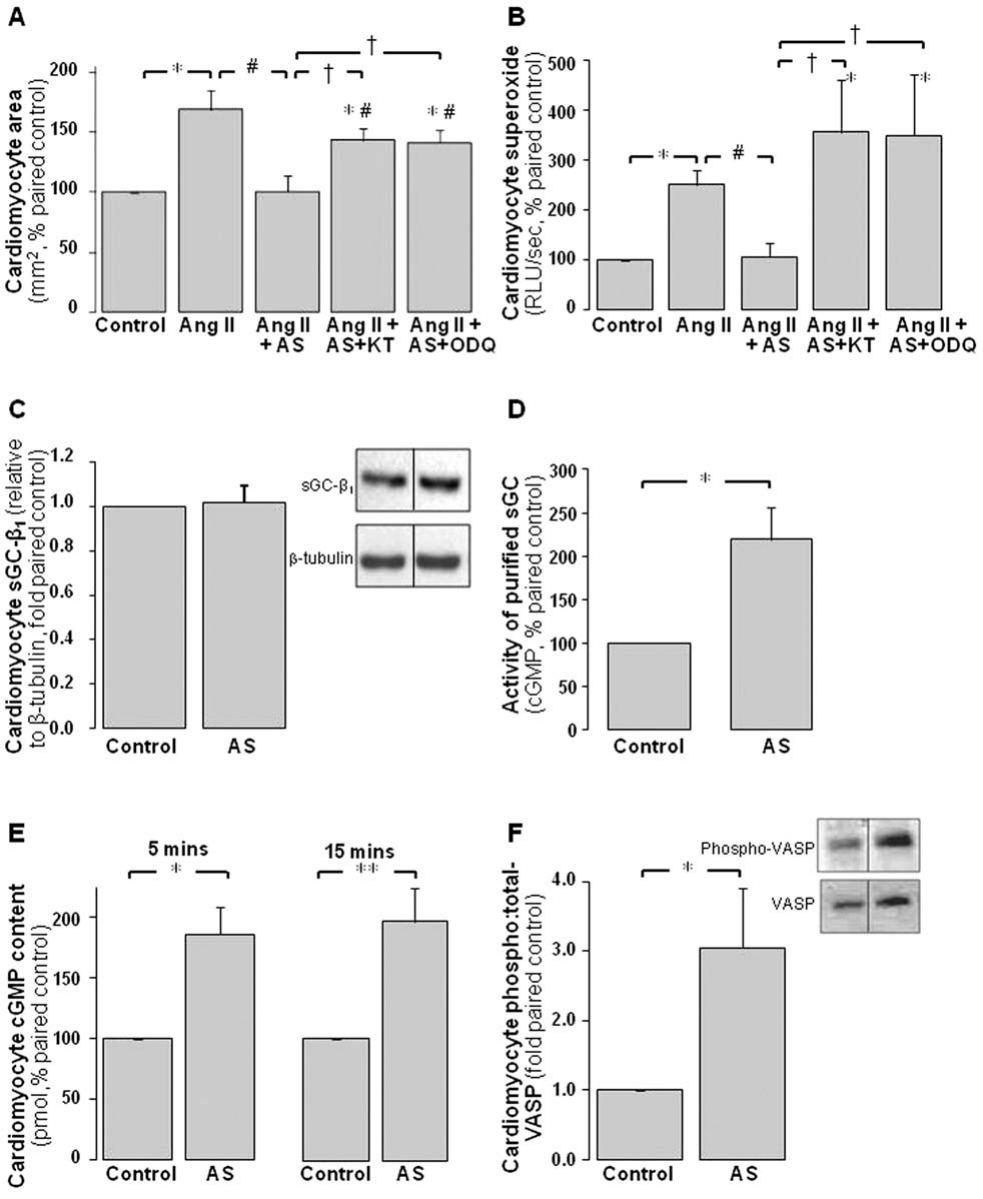
The antihypertrophic actions of Angeli's salt utilize sGC/cGMP signaling. **A** The antihypertrophic action of AS (1 µmol/L, added 4×/day over 48 h) on cell size is attenuated by the cGK-I inhibitor KT5823 (KT, 250 nmol/L) and by the sGC inhibitor ODQ (1 µmol/L, n = 5 myocyte preparations); and **B** the superoxide-suppressing actions of Angeli's salt are abolished by KT5823 and ODQ (n = 8 myocyte preparations). Furthermore, AS **C** does not affect cardiomyocyte sGC-β_1_ protein content (normalized to β-tubulin, n = 9 myocyte preparations), but acutely stimulates each of **D** purified sGC activity (over 10 min, n = 5), **E** cardiomyocyte cGMP accumulation (over 5 and 15 min, n = 3 and n = 11 myocyte preparations, respectively) and **F** the cGK-I biomarker, VASP phosphorylation (over 10 min, normalized to total VASP, n = 7 myocyte preparations). *P<0.05 and **P<0.005 vs control; ^#^P<0.05 vs Ang II alone; ^†^P<0.05 vs Ang II+AS.

Angeli's salt stimulates sGC to mediate its antihypertrophic and superoxide-suppressing actions in cardiomyocytes, as shown in Figure 4. The antihypertrophic effect of Angeli's salt is significantly attenuated by co-incubation with either the cGK-I inhibitor KT5823 (250 nmol/L) or the sGC inhibitor ODQ (1 µmol/L, Figure 4A, P<0.005 versus Ang II+Angeli's salt). Neither KT5823 nor ODQ significantly affect neonatal cardiomyocyte size, which was 104±12% and 101±14%, respectively (both n = 5, relative to paired controls). Similarly, co-incubation with either KT5823 or ODQ significantly attenuates the suppression of cardiomyocyte superoxide generation seen in the presence of Angeli's salt (Figure 4B, both P<0.05 versus Ang II+Angeli's salt). Neither KT5823, ODQ nor their vehicle alone affects this superoxide signal (results not shown). Further evidence of Angeli's salt sGC stimulation includes direct activation of purified sGC activity, 2.2±0.4-fold (10 min, n = 5); cardiomyocyte sGC protein content is however unchanged (β_1_-isoform, Figures 4C and 4D). As shown in Figure 4E, Angeli's salt also increases cardiomyocyte cGMP generation to 186±22% after 5 min (n = 3) and 201±27% after 15 min (n = 11). Cardiomyocyte content of both the cGK-I biomarker, phosphorylated VASP (3.1±0.8-fold at 10 min, Figure 4F, P<0.05), and cGK-I protein (2.2±0.7-fold at 48 h, n = 5 P = 0.1 vs control) also tend to increase with Angeli's salt.

### Angeli's salt inhibits endothelin-1-stimulated actions in neonatal cardiomyocytes

**Figure 5:**
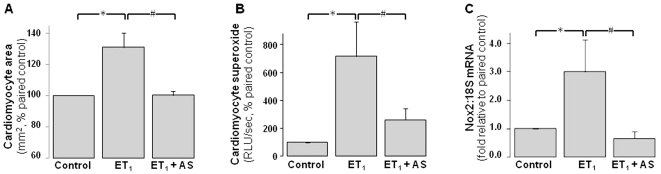
Angeli's salt also blunts endothelin-1 (ET_1_)-induced cardiomyocyte responses. AS (1 µmol/L, added 4×/day over 48 h) inhibits ET_1_ (60 nmol/L)-stimulated actions in neonatal cardiomyocytes. This is evident on **A** cardiomyocyte area (n = 3 myocyte preparations); **B** cardiomyocyte NADPH oxidase activity, on ET_1_-induced superoxide generation (lucigenin chemiluminescence, n = 7 myocyte preparations); and **C** cardiomyocyte Nox2 NADPH oxidase gene expression, induced by ET_1_ (n = 4 myocyte preparations). *P<0.05 vs control; ^#^P<0.05 vs ET_1_ alone.

Angeli's salt also attenuates responses to a second hypertrophic stimulus, endothelin-1 (ET_1_, 60 nmol/L). ET_1_ increases cardiomyocyte 2D area to 131±9% of control (Figure 5A, P<0.05), which is reduced to baseline levels Angeli's salt (Figure 5A). In addition, the ability of ET_1_ to increase NADPH-driven cardiomyocyte superoxide generation to 7.2±2.5 fold control (Figure 5B, P<0.05) and Nox2 expression (to 3.0±1.1-fold control, Figure 5C, P<0.05) is significantly attenuated by Angeli's salt (Figures 5B and 5C, both P<0.05 vs ET_1_ alone).

### BNP mimics the cGMP-dependent cardiomyocyte effects of Angeli's salt

**Figure 6:**
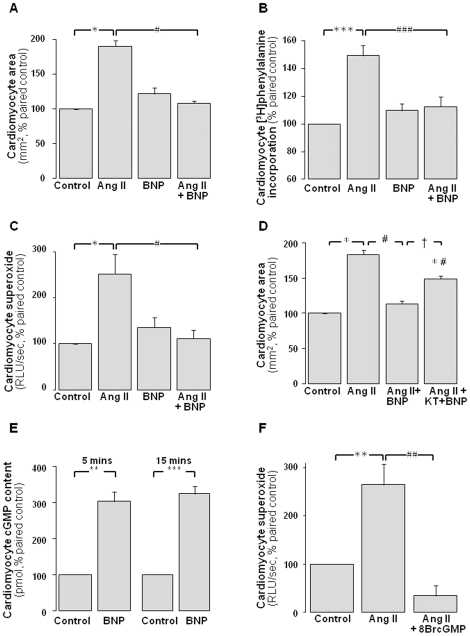
BNP mimics the antihypertrophic and cGMP-dependent cardiomyocyte effects of Angeli's salt. BNP (1 µmol/L, over 48 h) prevents Ang II (1 µmol/L)-stimulated cardiomyocyte hypertrophy. This is evident on both **A** cell size (n = 11 myocyte preparations); and **B** *de novo* protein synthesis (n = 9 myocyte preparations, P<0.001); in addition to **C** cardiomyocyte superoxide generation (n = 11 myocyte preparations). **D** These antihypertrophic actions of BNP are blocked by the cGK-I inhibitor KT5823 (KT, 250 nmol/L, n = 6 myocyte preparations). **E** BNP acutely stimulates cardiomyocyte cGMP accumulation, over 5 min and 15 min (both n = 5 myocyte preparations). **F** Furthermore, 8BrcGMP (1 mmol/L, n = 4) also mimics the ROS-suppressing actions of both AS and BNP. *P<0.05, **P<0.01 and ***P<0.001 vs control; ^#^P<0.05, ^##^P<0.01 and ^###^P<0.001 vs Ang II alone, ^†^P<0.05 vs Ang II+BNP.

Similar to Angeli's salt, the hypertrophic response to Ang II in neonatal rat cardiomyocytes is also prevented by the natriuretic peptide and particulate guanylyl cyclase (pGC) ligand, BNP (1 µmol/L). BNP reduces the Ang II- induced increase in 2D area from 190±9% to 108±3% of control (Figure 6A, P<0.05). Similarly, Ang II-induced [^3^H]phenylalanine incorporation was reduced from 149±8% to 113±7% by co-incubation with BNP (Figure 6B, P<0.001). The Ang II-induced increase in NADPH-driven cardiomyocyte superoxide generation was also blunted by BNP, from 2.5±0.4-fold to 1.1±0.2-fold control (Figure 6C, P<0.05); BNP alone did not significantly affect cardiomyocyte superoxide generation. Co-incubation with the cGK-I inhibitor KT5823 (250 nmol/L) significantly attenuates the antihypertrophic effect of BNP on cell size (Figure 6D, P<0.05 Ang II+BNP+KT5823 vs Ang II+BNP). Furthermore, BNP increases cardiomyocyte cGMP to 3.0±0.3-fold (P<0.01) and 3.2±0.2-fold paired control (P<0.001) after 5 and 15 min, respectively (Figure 6E). Lastly, 8BrcGMP (1 mmol/L) also mimics the ROS-suppressing actions of both Angeli's salt and BNP, as shown in Figure 6F.

### The actions of Angeli's salt are mediated via HNO

**Figure 7:**
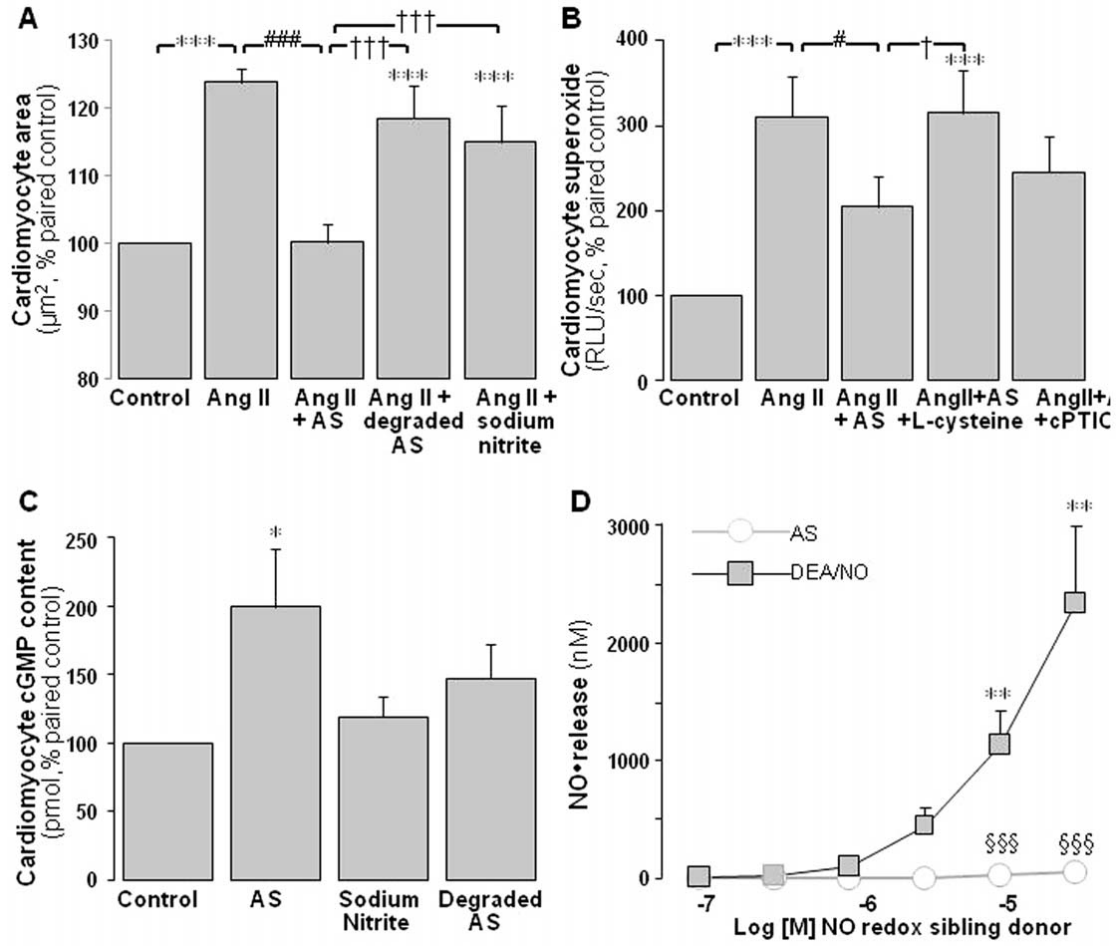
The actions of Angeli's salt are mediated via HNO. **A** Neither sodium nitrite (1 µmol/L, co-released by AS) nor degraded AS (1 µmol/L) significantly attenuate Ang II-stimulated cardiomyocyte hypertrophy on 2D area (n = 7 myocyte preparations). **B** The superoxide-suppressing actions of AS (1 µmol/L, added 4×/day over 48 h) are abolished by the HNO-selective scavenger L-cysteine (3 mmol/L) but are preserved in the presence of the NO^•^-selective scavenger carboxy-PTIO (cPTIO, 200 µmol/L, n = 6 myocyte preparations, P<0.001). **C** Neither sodium nitrite (1 µmol/L) nor degraded AS (1 µmol/L) significantly stimulate cGMP (both P = NS), in contrast to AS (n = 5 myocyte preparations). **D** Furthermore, the pure NO^•^ donor DEA/NO, but not AS, releases significant amounts of NO^•^ in a concentration-dependent manner over 0.1–30 µmol/L (n = 3). *P<0.05, **P<0.01 and ***P<0.001 vs control; ^#^P<0.05 and ^###^P<0.001 vs Ang II alone; ^†^P<0.05 and ^†††^P<0.001 vs Ang II+AS, ^§§§^P<0.001 AS vs same concentration of DEA-NO.

To confirm that the actions of Angeli's salt are mediated via HNO rather than nitrite (the other metabolite of Angeli's salt) or extracellular oxidation of HNO to NO^•^, we demonstrate that neither sodium nitrite nor degraded Angeli's salt (both 1 µmol/L) elicit significant inhibition of Ang II-stimulated cardiomyocyte hypertrophy (Figure 7A). The cardiomyocyte actions of intact Angeli's salt are completely prevented by the HNO-selective scavenger, L-cysteine (3 mmol/L, Figure 7B, P<0.05) but are unaffected by the NO^•^-selective scavenger, carboxy-PTIO (200 µmol/L). Furthermore, neither sodium nitrite nor degraded Angeli's salt (both 1 µmol/L) elicit significant impact on cardiomyocyte cGMP levels after 15 min, in direct contrast to paired cardiomyocytes treated with Angeli's salt (Figure 7C, both n = 5 cardiomyocyte preparations and P = NS vs control). Lastly, under our cell culture conditions, Angeli's salt fails to generate NO^•^, even at concentrations 30-fold higher than that used in the present study (Figure 7D). By contrast, DEA/NO (0.1–30 µmol/L) releases significant amounts of NO^•^ in a concentration-dependent manner (Figure 7D). Together these data indicate that the actions of Angeli's salt are solely mediated by HNO, without apparent contribution from either NO^•^ or nitrite.

## Discussion

The major finding to emerge from this study is the first evidence that an HNO donor potently blunts cardiomyocyte hypertrophy. Angeli's salt prevents all of the hypertrophic actions of Ang II in neonatal cardiomyocytes *in vitro*, including Ang II-induced increases in cell area, *de novo* protein synthesis and hypertrophic gene expression on β-myosin heavy chain analysis. Ang II-induced increases in cardiomyocyte NADPH oxidase expression (of the sarcolemmal Nox2 subunit) and activity (superoxide generation), as well as activation of p38MAPK, both implicated as triggers of the cardiomyocyte hypertrophic response *in vitro*, are also blunted by the HNO donor. The HNO donor is equally effective at blunting pro-hypertrophic and pro-oxidant responses, regardless of the hypertrophic stimulus (Ang II vs ET_1_). Further, no role for extracellular oxidation of HNO to NO^•^ , or of nitrite, in these actions was evident. The cGMP system is a powerful antihypertrophic mechanism in the heart [3], [23]–[28], and like NO^•^, the vascular actions of HNO appear to be mediated predominantly via the activation of sGC and a subsequent increase in cGMP [6]–[8], [12]. We now provide evidence that the HNO donor Angeli's salt elevates cardiomyocyte cGMP and directly activates sGC activity. Both the antihypertrophic and superoxide-suppressing effects of Angeli's salt are sensitive to both sGC and cGK-I inhibition. These findings confirm cGMP-dependence of these cardiac actions of HNO.

Angeli's salt is considered a classical HNO donor [3], [12]. It dissociates at physiological pH and temperature to yield HNO and nitrite (NO_2_^−^) [12]. Although nitrite is capable of stimulating sGC-dependent vasorelaxation [34], it is at least 15,000-fold less potent a vasodilator as Angeli's salt [6], [16], and only lowers blood pressure in rats *in vivo* at high concentrations (0.3–1.0 g/kg body weight) [34]. Based on our studies with sodium nitrite and degraded Angeli's salt; we now report that nitrite has negligible effects in cardiomyocytes and thus is unlikely to mediate the antihypertrophic actions of Angeli's salt. Under certain conditions (cell-free, in the absence of oxygen), higher concentrations of Angeli's salt than utilized in the present study (10 µmol/L) has been reported to also result in some generation of NO^•^ [16], likely via oxidation of HNO to NO^•^ by Cu^2+^ or Cu^2+^-containing enzymes (intracellular or extracellular) [12], [34], [35]. Whilst we cannot exclude the possibility of intra-cardiomyocyte oxidation of HNO to NO^•^ in our studies, we demonstrate that extracellular oxidation of HNO does not occur under our experimental conditions, as even at 30 µmol/L, no detectable NO^•^ is generated, in accordance with previous observations in the vasculature [6]. In addition, we show that cardiomyocyte responses to Angeli's salt are significantly attenuated by the selective HNO scavenger L-cysteine, but are completely unaffected by the NO^•^ scavenger carboxy-PTIO, analogous to its vasorelaxation responses [6], [7]. The sensitivity of Angeli's salt to the HNO scavenger lends further support to HNO (rather than nitrite or NO^•^) being the responsible entity for cardiomyocyte effects. Given that HNO (in contrast to NO^•^) is resistant to scavenging by ROS [12], [17]–[19], Angeli's salt retains its advantage over NO^•^ donors for limiting cardiomyocyte hypertrophy, particularly in settings of elevated ROS generation.

In the present study, we demonstrate that an HNO donor prevents cardiomyocyte hypertrophy via cGMP-dependent mechanisms that included suppression of NADPH oxidase. Such findings provide the first evidence that HNO exerts actions in the myocardium via the cGMP signaling pathway. As superoxide plays a pivotal role in triggering the hypertrophic response in the intact heart *in vivo*, and in cardiomyocytes *in vitro* [4], HNO/cGMP are an attractive antihypertrophic strategy [3], [21]. Our finding that the HNO donor Angeli's salt suppresses superoxide production and NADPH oxidase induction, is further evidence of HNO superoxide-suppressing actions in mammalian cells. Given that this action was mimicked by BNP and 8-BrcGMP, cGMP-generating agents thus appear to mediate their actions, at least in part, by suppressing cardiomyocyte NADPH oxidase expression and/or activity. The potential mechanism(s) of this action (e.g. cGK-mediated phosphorylation of Nox2) warrant further investigation.

Downstream of ROS, p38MAPK activation is a critical mediator of pathological cardiomyocyte hypertrophy induced by neurohumoral activation [2], [3], [36], [37]; cardiomyopathy often results. In contrast, Akt promotes physiological hypertrophy and prevents apoptosis [2], [38]. A similar role for ERK1/2 in cardiomyocyte survival and physiological hypertrophy has been proposed [2], [39]. Although Ang II- and ET_1_-induced cardiomyocyte ERK1/2 activation is often evident *in vitro* [2], [3], [30], [39], ERK1/2 does not contribute to pathological hypertrophy *in vivo* [40]. Interestingly, Angeli's salt selectively blunts Ang II-induced phosphorylation of p38MAPK. As shown in Figure 4, the actions of Angeli's salt are dependent on cGMP/cGK-I. MAPK phosphatase-1 (MKP-1) dephosphorylation of p38MAPK lies immediately downstream of cGK-I [3], [21]. It is thus possible that Angeli's salt enhances MKP-1 activity, to mediate the reduced p38MAPK phosphorylation observed here. Ang II-induced phosphorylation of both Akt and ERK1/2 however remained elevated after treatment with the HNO donor, despite normalization of three distinct parameters of cardiomyocyte hypertrophy (cardiomyocyte size, protein synthesis and hypertrophic gene expression). Given the cardioprotective properties of Akt [3], [38], the ability of HNO to inhibit cardiomyocyte hypertrophy in the face of preserved Akt signaling is a desirable trait.

The antihypertrophic effects of HNO are markedly attenuated by KT5823 or ODQ. Further, cardiomyocyte superoxide upregulation, a key trigger of cardiomyocyte hypertrophy [3], [21], [36], is completely prevented by both KT5823 and ODQ, leaving no residual cGMP-independent HNO actions. Although it is possible that the modest, apparently residual, component of the antihypertrophic actions of Angeli's salt that are not accounted for by KT5823 or ODQ could be due to calcitonin gene-related peptide (CGRP), this is probably unlikely. CGRP has been identified as a mediator of a component of HNO vasorelaxation [7]. The effects of CGRP on cardiac hypertrophy remain to be resolved however, with pro-hypertrophic effects observed *in vitro* [41] and antihypertrophic effects *in vivo* [42]. Further, superoxide is a key trigger of cardiomyocyte hypertrophy[3], [21], yet there was no parallel residual component of cardiomyocyte superoxide levels in Ang II-treated myocytes not prevented by KT5823 or ODQ. Robust sGC-independent cardiovascular actions of HNO linked to its reactivity with thiols include ryanodine receptors, protein N-nitrosation and S-glutathiolation, and activation of sarcoplasmic reticulum Ca^2+^-ATPase (SERCA) [10]–[12], [43]. Indeed, acute supra-pharmacological concentrations of Angeli's salt (up to 500-fold of those used here) directly activate SERCA, via S-glutathiolation at cysteine residue 674 [43] and disulfide bond formation on phospholamban (preventing its inhibition of SERCA) [44]. Thus, HNO-selective, thiol-mediated interactions independent of cGMP likely explain the inotropic and lusitropic actions of Angeli's salt [11], [43]. Our findings show in contrast that the antihypertrophic actions of HNO in contrast are critically dependent on cGMP.

Exploiting the cGMP antihypertrophic mechanism with chronic clinical use of traditional nitrovasodilators in the management of patients suffering hypertrophy/failing cardiac pathologies is limited, firstly by the phenomenon of “nitrate tolerance” [45]. In addition superoxide, generated in excess amounts in cardiac hypertrophy and failure, rapidly reacts with NO [3]. HNO donors offer considerable advantage over traditional NO^•^ donors as the redox siblings exhibit quite distinct pharmacology, both *in vitro* and *in vivo*. HNO donors neither exhibit cross-tolerance with organic nitrates (e.g. glyceryl trinitrate), nor do they induce tolerance to their own actions [20]. In addition, unlike NO^•^, HNO is resistant to scavenging by ROS [17]–[19]. Whilst the preference of HNO for Fe^3+^- versus Fe^2+^-heme groups [46] was initially thought to potentially permit HNO activation of the NO^•^-insensitive, oxidized form of sGC; this concept has now been refuted [35], [47]. Importantly, HNO elicits hemodynamic effects favorable in settings of cardiac remodeling and failure. This includes a marked positive inotropic effect that is both load- and reflex-independent, and persists even in failing myocardium *in vivo*. Moreover, HNO potentiates β-adrenergic inotropic responses in the failing heart [8], [9], [48]. These observations are all in direct contrast to conventional nitrovasodilators [9], [10], [17], [20].

### Limitations of the study

Our detailed investigation of the antihypertrophic actions of Angeli's salt and BNP, and the insights obtained into their mechanisms of action, were performed in a single cardiomyocyte strain and phenotype. The large majority of *in vitro* studies addressing cardiomyocyte hypertrophy similarly use neonatal rat cardiomyocyte preparations [21], [22], [28], [49], [50]. The antihypertrophic actions of Angeli's salt in adult cardiomyocytes may warrant further investigation. Cardiomyocytes isolated from adult mouse hearts are obtained in too few numbers, with too limited a timeframe of viability. We have previously demonstrated however that the antihypertrophic actions of BNP, like those of other cGMP-dependent antihypertrophic interventions, are observed in adult rat cardiomyocytes and/or the intact heart [22]–[26]. Hypertrophic responses in these settings are studied over a much shorter time-frame (2 h) and thus preclude assessment of changes in cell size.

### Concluding remarks

**Figure 8:**
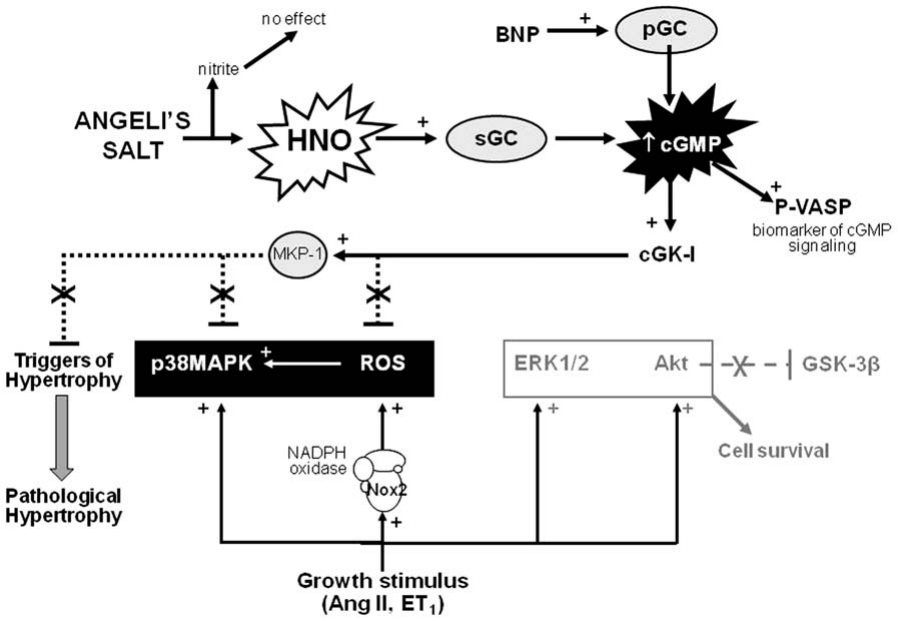
Mechanism of antihypertrophic action of HNO in cardiomyocytes. Angeli's salt utilizes HNO/sGC/cGMP/cGK-I signaling to suppress key triggers of the hypertrophic response, including expression and activity of NADPH oxidase (Nox2 subunit, a major source of reactive oxygen species, ROS) and activity of p38MAPK (the latter possibly as a result of enhanced activity of MAPK phosphatase-1, MKP-1). Activity of the cell survival kinase Akt (and its downstream target GSK-3β) remain intact in the presence of Angeli's salt. Cardiomyocyte hypertrophic responses across cell size, *de novo* protein synthesis and upregulated expression of β-myosin heavy chain are all ameliorated by HNO in the face of preserved cardiomyocyte ERK1/2 activation. Both the antihypertrophic and antioxidant actions of HNO are mediated via serial activation of sGC, cGMP production and cGK-I stimulation. Dashed lines indicate sites of inhibition. See text for references.

We now propose that stimulators of sGC that are not susceptible to ROS-mediated inactivation, and indeed suppress cardiomyocyte ROS generation, represent a superior approach to exploiting the antihypertrophic actions of the sGC/cGMP system in the heart. In conclusion, the present study suggests that HNO prevents acute cardiac hypertrophic responses (up to 48 h); cGMP-dependent suppression of cardiomyocyte NADPH oxidase and p38MAPK (key triggers of the hypertrophic response) likely contribute to these antihypertrophic actions (illustrated in Figure 8). Our findings indicate that longer-term studies of the antihypertrophic effects of this new class of agent *in vivo* are warranted. Given these potent antihypertrophic and superoxide-suppressing actions shown here, together with their established positive inotropic and vasodilatory actions, HNO donors may hence form the basis of more effective therapeutics for the clinical management of cardiac hypertrophy, alone or in combination with standard care.
